# Crystal structure of 3-(morpholin-4-yl)-1-phenyl-3-(pyridin-2-yl)propan-1-one

**DOI:** 10.1107/S2056989014026292

**Published:** 2015-01-01

**Authors:** F. M. Mashood Ahamed, M. Syed Ali Padusha, B. Gunasekaran

**Affiliations:** a PG and Research Dept of Chemistry, Jamal Mohamed College (Autonomous), Tiruchirappalli, Tamil Nadu 620 020, India; b Department of Physics & Nano Technology, SRM University, SRM Nagar, Kattankulathur, Kancheepuram Dist, Chennai 603 203 Tamil Nadu, India

**Keywords:** crystal structure, morpholin-4-yl, pyridin-2-yl, propan-1-one, biological activity

## Abstract

In the title compound C_18_H_20_N_2_O_2_, the morpholine ring adopts a chair conformation with the exocyclic N—C bond in an equatorial orientation. The N atom of the morpholine ring and the C atom of the carbonyl group are in an *anti* conformation about the central C—C bond [torsion angle = −162.92 (11)°] and the dihedral angle between the planes of the benzene ring and the pyridine ring is 83.30 (5)°. In the crystal, pairs of very weak C—H⋯π inter­actions link the mol­ecules into inversion dimers.

## Related literature   

For background to the biological activity of morpholine derivatives, see: Panneerselvam *et al.* (2009 ▸); Subhashini *et al.* (2013 ▸); Sawant *et al.* (2013 ▸); Dave & Sasaki (2006 ▸); For related structures, see: Chen *et al.* (2011 ▸); Meti *et al.* (2013 ▸);
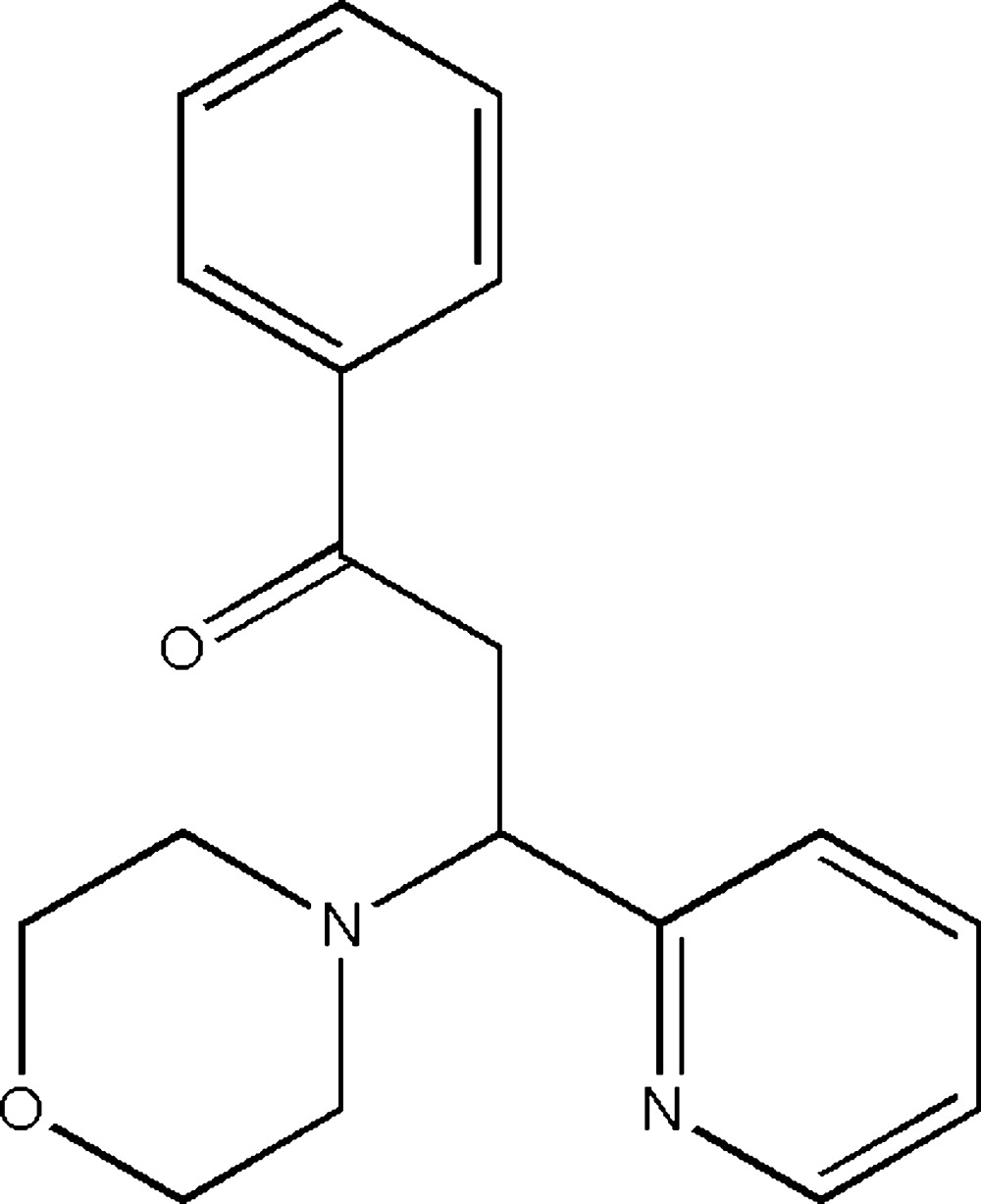


## Experimental   

### Crystal data  

C_18_H_20_N_2_O_2_
*M* *_r_* = 296.36
Orthorhombic, 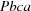

*a* = 12.4554 (6) Å
*b* = 8.2204 (4) Å
*c* = 30.6681 (17) Å
*V* = 3140.1 (3) Å^3^
*Z* = 8
Mo *K*α radiation
μ = 0.08 mm^−1^
*T* = 295 K
0.20 × 0.15 × 0.10 mm

### Data collection  

Bruker APEXII CCD diffractometer
Absorption correction: multi-scan (*SADABS*; Sheldrick, 1996 ▸) *T*_min_ = 0.954, *T*_max_ = 0.975
16093 measured reflections
3812 independent reflections
2547 reflections with *I* > 2σ(*I*)
*R*_int_ = 0.028

### Refinement  

*R*[*F* ^2^ > 2σ(*F* ^2^)] = 0.043
*wR*(*F* ^2^) = 0.111
*S* = 1.03
3812 reflections
199 parameters
H-atom parameters constrained
Δρ_max_ = 0.14 e Å^−3^
Δρ_min_ = −0.16 e Å^−3^

### 

Data collection: *APEX2* (Bruker, 2008 ▸); cell refinement: *SAINT* (Bruker, 2008 ▸); data reduction: *SAINT*; program(s) used to solve structure: *SHELXS97* (Sheldrick, 2008 ▸); program(s) used to refine structure: *SHELXL97* (Sheldrick, 2008 ▸); molecular graphics: *PLATON* (Spek, 2009 ▸); software used to prepare material for publication: *SHELXL97*.

## Supplementary Material

Crystal structure: contains datablock(s) I. DOI: 10.1107/S2056989014026292/hb7328sup1.cif

Structure factors: contains datablock(s) I. DOI: 10.1107/S2056989014026292/hb7328Isup2.hkl

Supporting information file. DOI: 10.1107/S2056989014026292/hb7328Isup3.cml

**Fig. 1.**
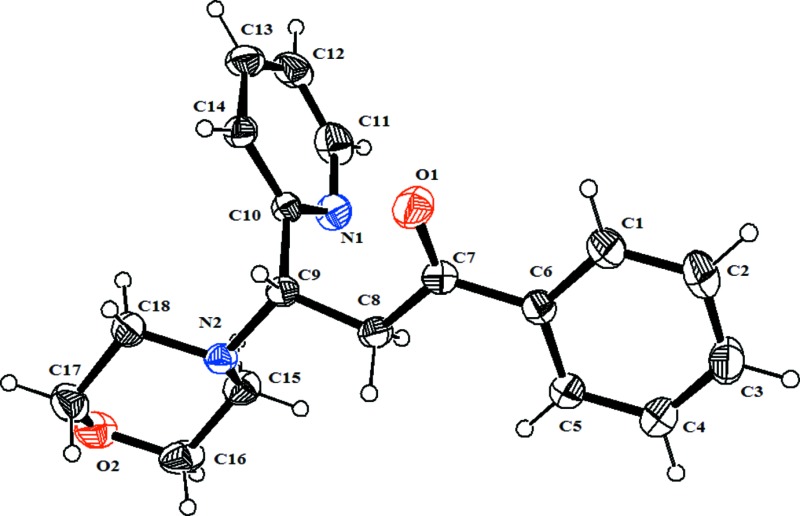
The molecular structure of (I), with 30% probability displacement ellipsoids for non-H atoms.

. DOI: 10.1107/S2056989014026292/hb7328fig1.tif
The mol­ecular structure of (I), with 30% probability displacement ellipsoids for non-H atoms.

CCDC reference: 1036843

Additional supporting information:  crystallographic information; 3D view; checkCIF report

## Figures and Tables

**Table 1:** Hydrogen-bond geometry (, ) *Cg*2 is the centroid of the C10C14/N1 ring.

*D*H*A*	*D*H	H*A*	*D* *A*	*D*H*A*
C2H2*Cg*2^i^	0.93	2.90	3.780(6)	159

Symmetry code: (i) 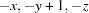.

## supplementary crystallographic information

### S1. Comment

Morpholines are six-membered heterocycles featuring both cyclic amine and ether functional group. These compounds possess important applications in pharmaceuticals and in industries (Panneerselvam *et al.*, 2009; Subhashini *et al.*, 2013). Chiral morpholine derivatives have found numerous applications in asymmetric synthesis as chiral auxiliaries as well as chiral ligands (Sawant *et al.*, 2013; Dave & Sasaki 2006).

The geometric parameters of the title molecule (Fig. 1) agree well with reported similar structure (Chen *et al.*, 2011; Meti *et al.*, 2013). The morpholine (N2/O2/C15—C18)ring adopts a chair conformation [Q = 0.5756 (3) Å, Θ = 179.09 (3)°, φ = 332.57 (5)°]. The phenyl ring makes a dihedral angles of 83.30 (5) ° with the pyridine ring. In the crystal, a weak C—H···π interaction is observed.

### S2. Experimental

To an ethanolic solution of acetophenone (3.0 ml, 0.025 mol) taken in a round bottom flask, morpholine (2.1 ml, 0.025 mol) and pyridine-2-carboldehyde (2.6 ml, 0.025 mol) were added. The reaction mixture was kept over a magnetic stirrer and stirred well in an ice cold condition for 3 hr. The colourless solid formed was filtered and washed several times with petroleum ether (40–60%). The crude solid obtained was dried and recrystallized using absolute alcohol. The recrystallized product was dried over vacuum. The yield is 78% and MP is 445 K.

### S3. Refinement

H atoms were positioned geometrically and refined using riding model with C—H = 0.93 Å and *U*_iso_(H) = 1.2Ueq(C) for aromatic C—H, C—H = 0.98 Å and *U*_iso_(H) = 1.2Ueq(C) for C—H, C—H = 0.97 Å and *U*_iso_(H) = 1.2Ueq(C) for C—H2,

### Crystal data

**Table:**

C_18_H_20_N_2_O_2_	*F*(000) = 1264
*M**_r_* = 296.36	*D*_x_ = 1.254 Mg m^−^^3^
Orthorhombic, *P**b**c**a*	Mo *K*α radiation, λ = 0.71073 Å
Hall symbol: -P 2ac 2ab	Cell parameters from 3812 reflections
*a* = 12.4554 (6) Å	θ = 1.3–28.4°
*b* = 8.2204 (4) Å	µ = 0.08 mm^−^^1^
*c* = 30.6681 (17) Å	*T* = 295 K
*V* = 3140.1 (3) Å^3^	Block, colourless
*Z* = 8	0.20 × 0.15 × 0.10 mm

### Data collection

**Table:**

Bruker APEXII CCD diffractometer	3812 independent reflections
Radiation source: fine-focus sealed tube	2547 reflections with *I* > 2σ(*I*)
Graphite monochromator	*R*_int_ = 0.028
Detector resolution: 0 pixels mm^-1^	θ_max_ = 28.4°, θ_min_ = 1.3°
ω and φ scans	*h* = −15→15
Absorption correction: multi-scan (*SADABS*; Sheldrick, 1996)	*k* = −10→10
*T*_min_ = 0.954, *T*_max_ = 0.975	*l* = −35→40
16093 measured reflections	

### Refinement

**Table:**

Refinement on *F*^2^	Primary atom site location: structure-invariant direct methods
Least-squares matrix: full	Secondary atom site location: difference Fourier map
*R*[*F*^2^ > 2σ(*F*^2^)] = 0.043	Hydrogen site location: inferred from neighbouring sites
*wR*(*F*^2^) = 0.111	H-atom parameters constrained
*S* = 1.03	*w* = 1/[σ^2^(*F*_o_^2^) + (0.0416*P*)^2^ + 0.6298*P*] where *P* = (*F*_o_^2^ + 2*F*_c_^2^)/3
3812 reflections	(Δ/σ)_max_ < 0.001
199 parameters	Δρ_max_ = 0.14 e Å^−^^3^
0 restraints	Δρ_min_ = −0.16 e Å^−^^3^

### Fractional atomic coordinates and isotropic or equivalent isotropic displacement parameters (Å^2^)

**Table:**

	*x*	*y*	*z*	*U*_iso_*/*U*_eq_	
C1	0.42802 (13)	−0.1732 (2)	0.02138 (5)	0.0604 (4)	
H1	0.5020	−0.1642	0.0182	0.073*	
C2	0.36782 (16)	−0.2413 (2)	−0.01152 (6)	0.0746 (5)	
H2	0.4013	−0.2768	−0.0369	0.089*	
C3	0.25900 (16)	−0.2571 (2)	−0.00707 (5)	0.0680 (5)	
H3	0.2186	−0.3050	−0.0291	0.082*	
C4	0.21022 (13)	−0.2020 (2)	0.02998 (6)	0.0653 (5)	
H4	0.1362	−0.2121	0.0330	0.078*	
C5	0.26935 (12)	−0.13170 (19)	0.06292 (5)	0.0529 (4)	
H5	0.2350	−0.0932	0.0878	0.063*	
C6	0.37980 (11)	−0.11808 (15)	0.05909 (4)	0.0412 (3)	
C7	0.44770 (10)	−0.04735 (16)	0.09421 (4)	0.0417 (3)	
C8	0.39381 (10)	0.03707 (17)	0.13181 (4)	0.0429 (3)	
H8A	0.3510	−0.0418	0.1477	0.051*	
H8B	0.3453	0.1190	0.1204	0.051*	
C9	0.47105 (9)	0.11758 (15)	0.16326 (4)	0.0364 (3)	
H9	0.5269	0.0372	0.1697	0.044*	
C10	0.52724 (10)	0.26260 (15)	0.14299 (4)	0.0368 (3)	
C11	0.51464 (16)	0.4990 (2)	0.10454 (6)	0.0666 (5)	
H11	0.4726	0.5746	0.0898	0.080*	
C12	0.62216 (16)	0.5287 (2)	0.10752 (6)	0.0678 (5)	
H12	0.6520	0.6215	0.0951	0.081*	
C13	0.68473 (13)	0.4190 (2)	0.12904 (5)	0.0610 (5)	
H13	0.7585	0.4344	0.1313	0.073*	
C14	0.63651 (11)	0.28526 (18)	0.14735 (5)	0.0452 (3)	
H14	0.6775	0.2099	0.1627	0.054*	
C15	0.33073 (11)	0.2736 (2)	0.20287 (5)	0.0532 (4)	
H15A	0.2787	0.2424	0.1809	0.064*	
H15B	0.3602	0.3787	0.1949	0.064*	
C16	0.27665 (12)	0.2851 (2)	0.24674 (6)	0.0662 (5)	
H16A	0.2195	0.3651	0.2453	0.079*	
H16B	0.2447	0.1808	0.2539	0.079*	
C17	0.43388 (14)	0.2128 (2)	0.28202 (5)	0.0632 (4)	
H17A	0.4040	0.1078	0.2898	0.076*	
H17B	0.4846	0.2440	0.3045	0.076*	
C18	0.49150 (11)	0.19882 (18)	0.23921 (4)	0.0462 (3)	
H18A	0.5247	0.3021	0.2320	0.055*	
H18B	0.5477	0.1176	0.2415	0.055*	
N1	0.46563 (10)	0.36867 (15)	0.12143 (4)	0.0526 (3)	
N2	0.41661 (8)	0.15290 (13)	0.20488 (4)	0.0394 (3)	
O1	0.54495 (8)	−0.05833 (15)	0.09246 (4)	0.0655 (3)	
O2	0.35010 (10)	0.32957 (14)	0.27995 (4)	0.0678 (3)	

### Atomic displacement parameters (Å^2^)

**Table:**

	*U*^11^	*U*^22^	*U*^33^	*U*^12^	*U*^13^	*U*^23^
C1	0.0564 (9)	0.0742 (11)	0.0506 (9)	−0.0007 (8)	0.0083 (8)	−0.0126 (8)
C2	0.0842 (13)	0.0925 (14)	0.0470 (10)	−0.0019 (11)	0.0067 (9)	−0.0220 (9)
C3	0.0815 (12)	0.0728 (11)	0.0497 (9)	−0.0123 (10)	−0.0134 (9)	−0.0073 (9)
C4	0.0567 (9)	0.0784 (12)	0.0607 (10)	−0.0166 (9)	−0.0049 (8)	−0.0085 (9)
C5	0.0495 (9)	0.0615 (9)	0.0477 (8)	−0.0098 (7)	0.0030 (7)	−0.0074 (7)
C6	0.0473 (7)	0.0366 (7)	0.0399 (7)	−0.0009 (6)	0.0012 (6)	0.0008 (6)
C7	0.0410 (7)	0.0392 (7)	0.0449 (8)	0.0005 (6)	0.0027 (6)	0.0003 (6)
C8	0.0379 (7)	0.0446 (7)	0.0461 (8)	−0.0049 (6)	0.0040 (6)	−0.0039 (6)
C9	0.0328 (6)	0.0363 (7)	0.0402 (7)	0.0024 (5)	0.0011 (5)	0.0002 (6)
C10	0.0376 (7)	0.0382 (7)	0.0346 (6)	−0.0004 (5)	0.0020 (5)	−0.0025 (5)
C11	0.0910 (13)	0.0498 (9)	0.0589 (10)	−0.0004 (9)	−0.0032 (9)	0.0166 (8)
C12	0.0926 (13)	0.0549 (10)	0.0559 (10)	−0.0279 (10)	0.0183 (9)	0.0017 (8)
C13	0.0555 (9)	0.0663 (10)	0.0613 (10)	−0.0229 (8)	0.0155 (8)	−0.0162 (9)
C14	0.0389 (7)	0.0512 (8)	0.0456 (8)	−0.0027 (6)	0.0030 (6)	−0.0067 (7)
C15	0.0404 (7)	0.0569 (9)	0.0624 (10)	0.0062 (7)	0.0048 (7)	−0.0090 (8)
C16	0.0500 (9)	0.0641 (10)	0.0844 (12)	−0.0057 (8)	0.0237 (9)	−0.0198 (9)
C17	0.0810 (11)	0.0612 (10)	0.0473 (9)	−0.0109 (9)	0.0080 (8)	−0.0065 (8)
C18	0.0483 (8)	0.0451 (8)	0.0453 (8)	−0.0029 (7)	−0.0002 (6)	−0.0014 (6)
N1	0.0530 (7)	0.0496 (7)	0.0553 (7)	0.0018 (6)	−0.0067 (6)	0.0125 (6)
N2	0.0355 (5)	0.0409 (6)	0.0419 (6)	−0.0012 (5)	0.0040 (5)	−0.0024 (5)
O1	0.0412 (6)	0.0847 (9)	0.0707 (8)	0.0064 (5)	0.0016 (5)	−0.0247 (6)
O2	0.0733 (7)	0.0648 (7)	0.0654 (7)	−0.0114 (6)	0.0215 (6)	−0.0235 (6)

### Geometric parameters (Å, º)

**Table:**

C1—C2	1.376 (2)	C11—N1	1.338 (2)
C1—C6	1.380 (2)	C11—C12	1.364 (3)
C1—H1	0.9300	C11—H11	0.9300
C2—C3	1.368 (3)	C12—C13	1.362 (2)
C2—H2	0.9300	C12—H12	0.9300
C3—C4	1.366 (2)	C13—C14	1.373 (2)
C3—H3	0.9300	C13—H13	0.9300
C4—C5	1.377 (2)	C14—H14	0.9300
C4—H4	0.9300	C15—N2	1.4604 (17)
C5—C6	1.3852 (19)	C15—C16	1.508 (2)
C5—H5	0.9300	C15—H15A	0.9700
C6—C7	1.4878 (19)	C15—H15B	0.9700
C7—O1	1.2159 (15)	C16—O2	1.417 (2)
C7—C8	1.5039 (19)	C16—H16A	0.9700
C8—C9	1.5146 (18)	C16—H16B	0.9700
C8—H8A	0.9700	C17—O2	1.419 (2)
C8—H8B	0.9700	C17—C18	1.500 (2)
C9—N2	1.4741 (16)	C17—H17A	0.9700
C9—C10	1.5157 (18)	C17—H17B	0.9700
C9—H9	0.9800	C18—N2	1.4565 (17)
C10—N1	1.3365 (17)	C18—H18A	0.9700
C10—C14	1.3803 (17)	C18—H18B	0.9700
			
C2—C1—C6	120.73 (15)	C13—C12—C11	118.42 (15)
C2—C1—H1	119.6	C13—C12—H12	120.8
C6—C1—H1	119.6	C11—C12—H12	120.8
C3—C2—C1	120.36 (16)	C12—C13—C14	118.55 (15)
C3—C2—H2	119.8	C12—C13—H13	120.7
C1—C2—H2	119.8	C14—C13—H13	120.7
C4—C3—C2	119.47 (16)	C13—C14—C10	119.99 (15)
C4—C3—H3	120.3	C13—C14—H14	120.0
C2—C3—H3	120.3	C10—C14—H14	120.0
C3—C4—C5	120.76 (15)	N2—C15—C16	109.39 (13)
C3—C4—H4	119.6	N2—C15—H15A	109.8
C5—C4—H4	119.6	C16—C15—H15A	109.8
C4—C5—C6	120.18 (14)	N2—C15—H15B	109.8
C4—C5—H5	119.9	C16—C15—H15B	109.8
C6—C5—H5	119.9	H15A—C15—H15B	108.2
C1—C6—C5	118.48 (13)	O2—C16—C15	111.65 (12)
C1—C6—C7	119.19 (12)	O2—C16—H16A	109.3
C5—C6—C7	122.33 (12)	C15—C16—H16A	109.3
O1—C7—C6	120.35 (13)	O2—C16—H16B	109.3
O1—C7—C8	120.85 (12)	C15—C16—H16B	109.3
C6—C7—C8	118.80 (11)	H16A—C16—H16B	108.0
C7—C8—C9	113.98 (10)	O2—C17—C18	111.37 (13)
C7—C8—H8A	108.8	O2—C17—H17A	109.4
C9—C8—H8A	108.8	C18—C17—H17A	109.4
C7—C8—H8B	108.8	O2—C17—H17B	109.4
C9—C8—H8B	108.8	C18—C17—H17B	109.4
H8A—C8—H8B	107.7	H17A—C17—H17B	108.0
N2—C9—C8	110.20 (10)	N2—C18—C17	110.24 (12)
N2—C9—C10	114.37 (10)	N2—C18—H18A	109.6
C8—C9—C10	112.07 (11)	C17—C18—H18A	109.6
N2—C9—H9	106.6	N2—C18—H18B	109.6
C8—C9—H9	106.6	C17—C18—H18B	109.6
C10—C9—H9	106.6	H18A—C18—H18B	108.1
N1—C10—C14	121.74 (13)	C10—N1—C11	116.89 (13)
N1—C10—C9	116.80 (11)	C18—N2—C15	108.87 (11)
C14—C10—C9	121.45 (12)	C18—N2—C9	112.48 (10)
N1—C11—C12	124.40 (17)	C15—N2—C9	115.74 (11)
N1—C11—H11	117.8	C16—O2—C17	109.40 (12)
C12—C11—H11	117.8		
			
C6—C1—C2—C3	−0.8 (3)	N1—C11—C12—C13	0.0 (3)
C1—C2—C3—C4	1.2 (3)	C11—C12—C13—C14	−1.1 (2)
C2—C3—C4—C5	−0.3 (3)	C12—C13—C14—C10	1.3 (2)
C3—C4—C5—C6	−0.9 (3)	N1—C10—C14—C13	−0.4 (2)
C2—C1—C6—C5	−0.5 (2)	C9—C10—C14—C13	−179.19 (13)
C2—C1—C6—C7	179.11 (16)	N2—C15—C16—O2	59.10 (17)
C4—C5—C6—C1	1.3 (2)	O2—C17—C18—N2	−58.71 (16)
C4—C5—C6—C7	−178.26 (14)	C14—C10—N1—C11	−0.6 (2)
C1—C6—C7—O1	−10.1 (2)	C9—C10—N1—C11	178.23 (13)
C5—C6—C7—O1	169.48 (15)	C12—C11—N1—C10	0.8 (3)
C1—C6—C7—C8	170.08 (13)	C17—C18—N2—C15	57.48 (15)
C5—C6—C7—C8	−10.3 (2)	C17—C18—N2—C9	−172.87 (12)
O1—C7—C8—C9	5.6 (2)	C16—C15—N2—C18	−57.27 (15)
C6—C7—C8—C9	−174.59 (11)	C16—C15—N2—C9	174.89 (11)
C7—C8—C9—N2	−162.92 (11)	C8—C9—N2—C18	168.06 (11)
C7—C8—C9—C10	68.50 (15)	C10—C9—N2—C18	−64.62 (14)
N2—C9—C10—N1	−79.35 (15)	C8—C9—N2—C15	−65.92 (14)
C8—C9—C10—N1	47.00 (15)	C10—C9—N2—C15	61.40 (14)
N2—C9—C10—C14	99.47 (14)	C15—C16—O2—C17	−58.86 (18)
C8—C9—C10—C14	−134.18 (13)	C18—C17—O2—C16	58.37 (16)

### Hydrogen-bond geometry (Å, º)

Cg2 is the centroid of the C10–C14/N1 ring.

**Table:**

*D*—H···*A*	*D*—H	H···*A*	*D*···*A*	*D*—H···*A*
C2—H2···*Cg*2^i^	0.93	2.90	3.780 (6)	159

Symmetry code: (i) −*x*, −*y*+1, −*z*.

## References

[bb1] Bruker (2008). *APEX2* and *SAINT*. Bruker AXS Inc., Madison, Wisconsin, USA.

[bb2] Chen, X.-Y., Zhao, M.-M., Qian, X. & Hou, S.-G. (2011). *Acta Cryst.* E**67**, o3484. 10.1107/S160053681104997X PMC3239108 22199956

[bb3] Dave, R. & Sasaki, N. A. (2006). *Tetrahedron Asymmetry*, **17**, 388–401.

[bb4] Meti, G. Y., Kamble, R. R., Ravi, A. J., Arunkashi, H. K. & Devarajegowda, H. C. (2013). *Acta Cryst.* E**69**, o129. 10.1107/S1600536812050957 PMC3588350 23476389

[bb5] Panneerselvam, P., Priya, M., Gnanarupa, , Kumar, N., Ramesh, & Saravanan, G. (2009). *Indian J. Pharm. Sci.* **71**, 428–432. 10.4103/0250-474X.57292 PMC2865815 20502549

[bb6] Sawant, R. T., Stevenson, J., Odell, L. R. & Arvidsson, P. I. (2013). *Tetrahedron Asymmetry*, **24**, 134–141.

[bb7] Sheldrick, G. M. (1996). *SADABS*. University of Göttingen, Germany.

[bb8] Sheldrick, G. M. (2008). *Acta Cryst.* A**64**, 112–122. 10.1107/S0108767307043930 18156677

[bb9] Spek, A. L. (2009). *Acta Cryst.* D**65**, 148–155. 10.1107/S090744490804362X PMC2631630 19171970

[bb10] Subhashini, N. J. P., Amanaganti, J., Boddu, L. & Acharya Nagarjuna, P. (2013). *J. Chem. Pharm. Res.* **5**, 140–147.

