# Supplementary figures and images for: Crystal structure of 3-(morpholin-4-yl)-1-phenyl-3-(pyridin-2-yl)propan-1-one
Source: Acta Crystallogr E Crystallogr Commun. 2015 Jan 1;71(Pt 1):o24–5. doi: 10.1107/S2056989014026292 (PMC4331866; doi:10.1107/S2056989014026292)

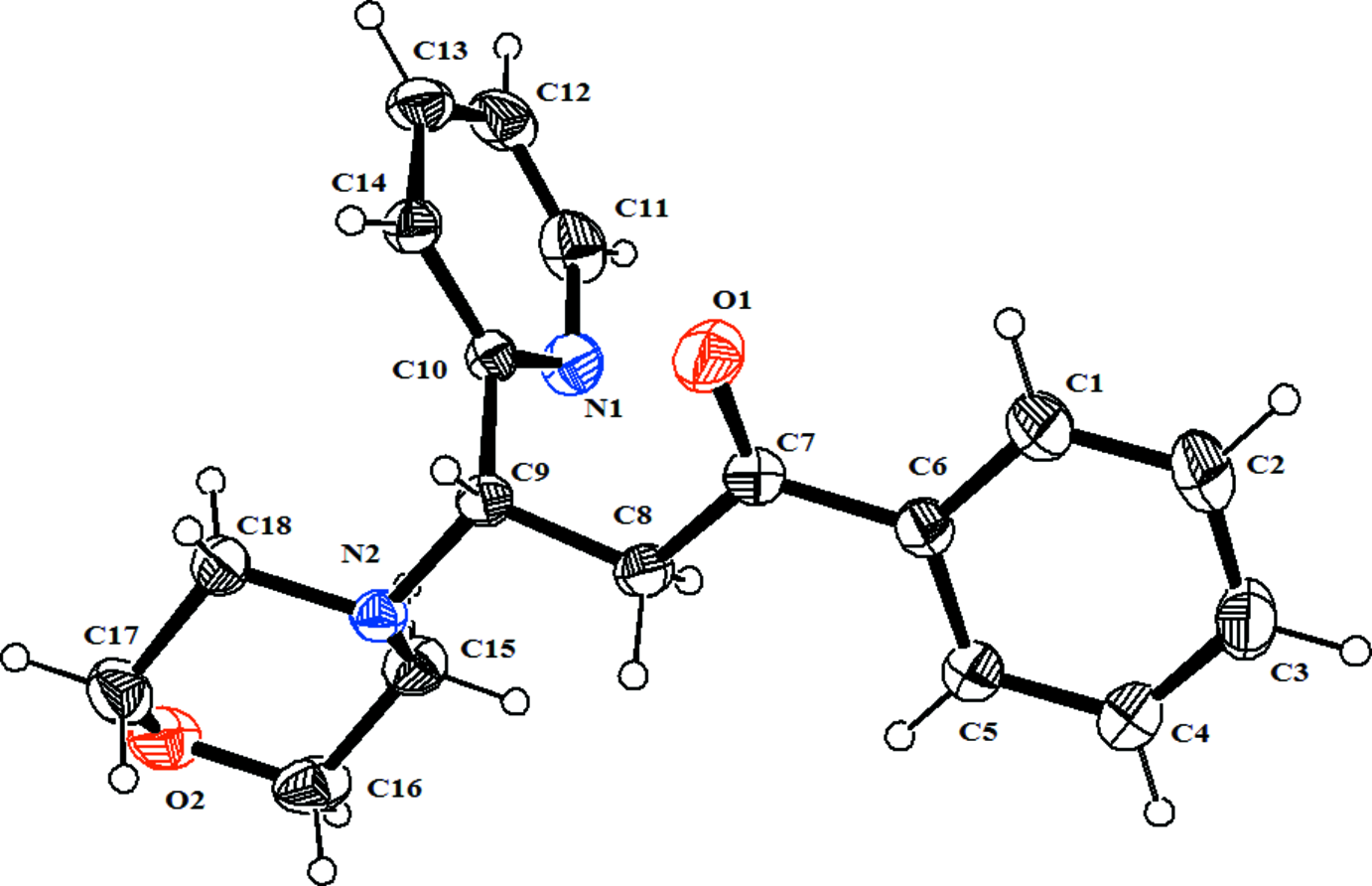

Supplement: Supplementary file 4 [file e-71-00o24-fig1.tif]
